# Resonant Tunneling Nanostructures: Eliminating Current Saturation on Negative Differential Conductivity Region in Compact Dissipative Simulations

**DOI:** 10.3390/nano15020100

**Published:** 2025-01-10

**Authors:** Natalia Vetrova, Evgeny Kuimov, Sergey Meshkov, Vladimir Sinyakin, Mstislav Makeev, Vasiliy Shashurin

**Affiliations:** Research Institute of Radio Electronics and Laser Technology of Bauman Moscow State Technical University, 105005 Moscow, Russia; kuimov@bmstu.ru (E.K.); meschkow@bmstu.ru (S.M.); v.sinyakin@gmail.com (V.S.); m.makeev@bmstu.ru (M.M.); schashurin@bmstu.ru (V.S.)

**Keywords:** resonant tunneling, heterostructures, compact modeling

## Abstract

A solution to the problem of resonant tunneling current saturation is proposed. This problem does not allow, within the traditional compact models, a correct qualitative and quantitative analysis to be carried out of the volt-ampere characteristics of double-barrier heterostructures. The reason for this problem is the asymptotic behavior of the function describing the structure transparency, so a non-saturating compact model was proposed to solve the problem of current transfer analysis in the region of negative differential conductivity. Validation of the proposed model confirmed its adequacy without losing the ability to analyze current transfer processes. This makes the developed compact model effective for simulating the operation of a wide range of devices with a resonant tunneling diode as a nonlinear element, regardless of the position of the operating point.

## 1. Introduction

Today, in the development of radio electronics, one can identify trends towards increasing operating frequencies and powers of processed signals. The decisive influence on the key parameters of radio electronic functional units in the receiving and transmitting path (frequency mixers and multipliers, power detectors, signal generators, etc.) is exerted by the electrical characteristics of nonlinear elements, primarily the volt-ampere characteristics (VACs) of diodes and transistors. The transition to the new electronic components based on the use of such quantum-mechanical phenomena as de Broglie wave interference, the tunnel effect, resonant tunneling [1,2,3,4,5,6,7,8,9] and others, allows electronic devices to be designed that meet increasing requirements for functional characteristics.

An example of such an electronic component is a resonant tunneling diode (RTD), which is of interest in terms of the VAC nonlinearity. Studies in recent years [10,11,12,13,14,15,16,17] show that due to a higher response speed than Schottky and tunnel diodes and the ability to vary the VAC, the use of an RTD as a nonlinear element allows improvement in the performance of a wide range of electronic devices, for example, expanding the dynamic range of radio frequency mixers, the efficiency of rectifiers, increasing the operating frequency of signal generators up to the THz range, etc.

However, the introduction of RTDs into modern industry is complicated by a number of reasons, including problems of technological reproducibility and ensuring a given VAC shape at the RTD design stage, which is limited by the capabilities of current transfer models in RTDs. The current transfer models used today belong to the theoretical class, the so-called models “from first principles” [18,19], which are structurally complex systems of integral-differential equations. Therefore, the algorithms for calculating the RTD VAC based on theoretical class models have an unreasonably high spatial and temporal complexity. At the same time, since the design optimization process requires modeling hundreds and thousands of RTD design options, theoretical class models are not suitable for the tasks of designing radio electronic devices.

Currently, there are a number of “compact” models of current transport in heterostructure devices [20,21,22,23,24,25,26,27,28,29,30], built on the basis of theoretical class models. However, the applicability of existing compact models is limited by a number of circumstances, one of which is the limitation of the acceptable reliability of the VAC prediction by a narrow voltage range. Particularly problematic is the section of negative differential conductivity, where it is quite difficult to obtain even qualitative correspondence with the experiment. Thus, the task of developing a compact model of the RTD VAC for design and technological optimization of the RTD seems relevant, allowing, within the compact representation, estimates of the RTD VAC in various regions to be carried out with a prediction accuracy acceptable for engineering calculations.

## 2. Materials and Methods

In the coherent transport approximation, the current density through a resonant tunneling structure (RTS) is described by the well-known Tsu-Esaki formula [31]

(1) $$J_{RT}=\frac{q}{\pi \hbar }\int_{0}^{\infty }T\left(E\right)D\left(E\right)dE$$

where $J_{RT}$—resonant tunneling current density, q—elementary charge, ℏ—Dirac constant, E—transverse component of the electron energy, $T\left(E\right)$—tunnel transparency coefficient and $D\left(E\right)$—supply function, which is defined as

(2) $$D\left(E\right)=f_{2D}\left(E\right)-f_{2D}\left(E+qV\right)$$

where V—external voltage and $f_{2D}\left(E\right)$—distribution function of a two-dimensional electron gas

(3) $$f_{2D}\left(E\right)=\frac{mkT}{2\pi \hbar^{2}}\ln\left(1+e^{\frac{E_{F}-E}{kT}}\right)$$

where m—effective mass of electrons in reservoirs, k—Boltzmann constant, T—absolute temperature and $E_{F}$—Fermi level at source.

Formula (1) is the basis for a large number of quantum-mechanical models [24,26,28,31], from the analysis of which, in terms of applicability to design problems, the need to move to the class of compact models is obvious. Indeed, even without taking into account the scattering processes, the use of a quantum-mechanical charge-transfer model is difficult both for a qualitative analysis of current transfer processes in order to identify the main factors affecting the VAC shape in different regions, and for quantitative VAC estimates in the procedure for optimizing the RTD design, where multiple calculations of VAC are required, since such a quantum-mechanical model has too high a computational complexity. Therefore, for the analysis of current transfer processes and practical calculations, in general, it is preferable to use compact models of the RTS VAC.

**Figure 1:**
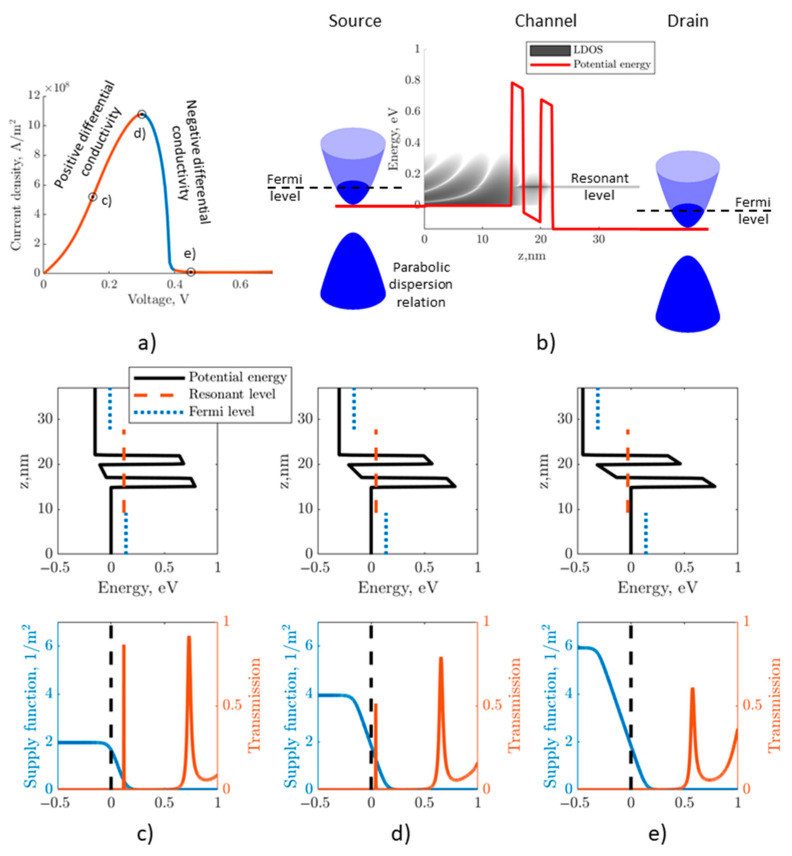
Demonstration of the mechanism for forming the RTS VAC. (**a**) Typical calculation of the RTS VAC using formula (1) with the points indicated for which the graphs of the supply functions (orange line) and the tunnel transparency coefficient (blue line) (**c**–**e**) are constructed. (**b**) Band diagram and local density of states of electrons in the RTD channel.

Compact models are constructed on the basis of theoretical class models taking into account the features of current transfer revealed in the analysis of the results of numerical and full-scale experiments. Features of current transfer in RTS are associated with the behavior of resonant levels—metastable states in quantum wells, which are the result of the interference of wave functions, manifested in the resonant nature of the tunnel transparency coefficient. Based on the results of current transfer modeling using the “verification” basis model [18,19], the following conclusions were made about the properties of resonant levels (Figure 1, RTS based on GaAs/AlAs-heterojunctions, spacer thickness: 15 nm, i-GaAs layer thickness: 3 nm, i-AlAs layer thickness: 2 nm, Fermi level: 14 meV):

1. The current density and electron concentration in the quantum well are determined by electrons with energy in the vicinity of the lowest resonant level formed in the RTS quantum well;
2. The width of the resonant levels is negligibly small compared to the thermal energy and does not depend on the voltage on the diode. Thus, for all practically significant RTD designs (barrier width range up to 8–10 monolayers, well width up to 20 monolayers), the width of the resonant levels is about 0.01 meV and lower, while at the lowest operating temperatures (77 K), the thermal energy is 7 meV;
3. The tunnel transparency coefficient and the local density of states depending on the energy in the vicinity of the resonant levels can be approximated by a function proportional to the Lorentz distribution function.

The listed features of the resonant level parameters and their dependence on external voltage are the key factor determining the properties of charge transfer processes in heterostructures and form the basis of a large number of current transfer compact models as assumptions, with the help of which formula (1) is reduced to a compact representation. In this way, for example, the Schulman model [32,33,34,35] was obtained, which is both the most common compact model of current transfer in RTDs and the basis for more complex models of compact representation; therefore, this model will be considered in more detail below.

Within the Schulman model, the resonant tunneling current density, obtained from the Tsu-Esaki formula using the approximation of the tunnel transparency coefficient by the sum of Lorentzian functions

(4) $$T\left(E\right)=\sum_{n=1}^{N}\frac{\Gamma_{n}^{2}}{{\left(E-\varepsilon_{n}\right)}^{2}+\Gamma_{n}^{2}}$$

can be represented as a dependence

(5) $$J_{RT}\left(V\right)=\frac{q}{\pi \hbar }\sum_{n=1}^{N}\int_{0}^{\infty }\frac{\Gamma_{n}^{2}}{{\left(E-\varepsilon_{n}\right)}^{2}+\Gamma_{n}^{2}}D\left(E\right)dE$$

where $\varepsilon_{n}$—resonant level energy, $\Gamma_{n}$—half-width of the resonant level and N—number of resonant levels.

In a first approximation, it is assumed that the energy of the resonant level is proportional to the external voltage

(6) $$\varepsilon_{n}=\varepsilon_{0n}-qk_{V}V$$

where $\varepsilon_{0n}$—resonant level energy in the absence of an external field and $k_{V}$—asymmetry coefficient (${0<k}_{V}<1$).

The resonant level width is determined by the design parameters of the RTD, namely: the thickness of the barrier layers and well layers; the magnitude of the discontinuity of the conduction band bottom at the boundary of the heterolayers in the channel. In the case where the second of the listed assumptions is fulfilled, the width of the resonant levels is sufficiently small (~10^−5^ eV) compared to the scales on which the change in the supply function $D\left(E,V\right)$ is noticeable, so $D\left(E,V\right)$ can be considered constant in the vicinity of the resonant level. Then,

(7) $$J_{RT}\left(V\right)=\frac{q}{\pi \hbar }\sum_{n=1}^{N}D\left(\varepsilon_{n}\right)\int_{0}^{\infty }\frac{\Gamma_{n}^{2}}{{\left(E-\varepsilon_{n}\right)}^{2}+\Gamma_{n}^{2}}dE=\frac{q}{\hbar }\sum_{n=1}^{N}\Gamma_{n}D\left(\varepsilon_{n}\right)\frac{1}{\pi }\int_{-\frac{\varepsilon_{n}}{\Gamma_{n}}}^{\infty }\frac{1}{y^{2}+1}dy$$

Let us introduce the following function:

(8) $$F\left(x\right)=\frac{1}{\pi }\int_{-x}^{\infty }\frac{1}{y^{2}+1}dy=\frac{1}{\pi }\left(\frac{\pi }{2}+\mathrm{arctg}x\right)=\frac{1}{\pi }\mathrm{arctg}x+\frac{1}{2}$$

which has the meaning of a fraction of the resonant level width involved in current transfer. It is obvious that this fraction should decrease when the resonant level passes through the bottom of the conduction band at the source.

Then, the expression for the resonant tunneling current density can be written as

(9) $$J_{RT}\left(V\right)=\frac{q}{\hbar }\sum_{n=1}^{N}\Gamma_{n}D\left(\varepsilon_{n}\right)F\left(\frac{\varepsilon_{n}}{\Gamma_{n}}\right)$$

Thus, the Schulman model defines the density of the resonant tunneling current through the RTD as a function of voltage (9). Just as in the case of the quantum-mechanical model, to improve the correspondence of the calculation results to the experiment, scattering processes are introduced into the model.

**Figure 2:**
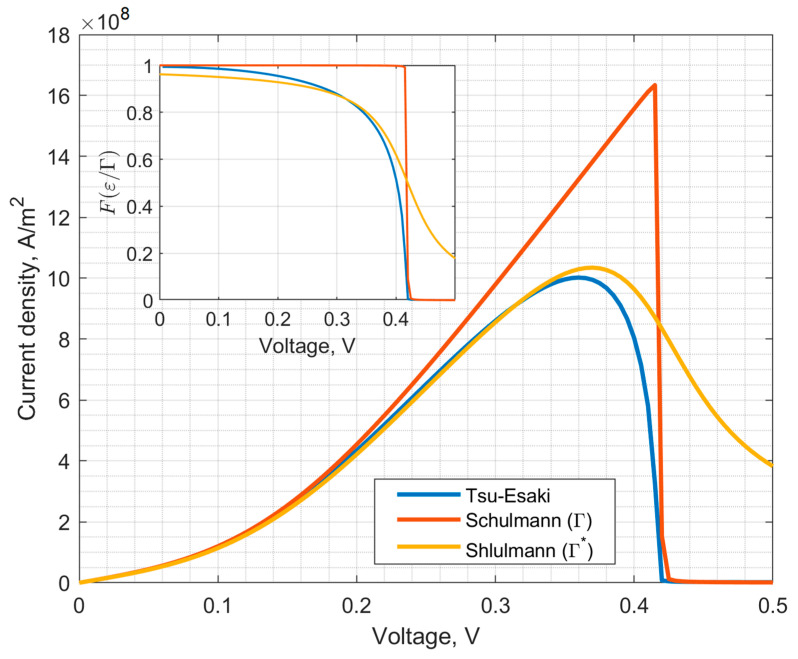
Calculation of the RTS VAC using the Schulman model and the Tsu-Esaki formula and on inset—function $F\left(\frac{\varepsilon }{\Gamma }\right)$ vs. voltage for one-level channel.

Thus, the dependence of the current density on the voltage is determined by the set of parameters $\left\{\varepsilon_{n},\Gamma_{n},k_{V},E_{F}\right\}$. A comparison of the results of calculating the current density using the Schulman model and the Tsu-Esaki formula is shown in Figure 2. Simulated RTS parameters were the same as for calculations of Figure 1. Note that the Schulman model gives overestimated peak current values compared to the Tsu-Esaki formula, as well as a different VAC curvature in the near-peak region. This is explained by the fact that when deriving the dependence of the current density on the voltage (9), it is assumed that the maximum value of the tunnel transparency coefficient $T\left(\varepsilon_{n}\right)=1$, although it follows from quantum-mechanical calculations that $T\left(\varepsilon_{n}\right)$ is generally not constant and depends on the voltage on the RTS. In order not to complicate the model, the dependence of $T\left(\varepsilon_{n}\right)$ on the voltage is effectively taken into account using the function $F\left(x\right)$. To do this, the parameter $\Gamma_{n}$ in $F\left(\frac{\varepsilon_{n}}{\Gamma_{n}}\right)$ is replaced by the parameter $\Gamma_{n}^{*}$, which is not related to broadening, and it is selected in such a way as to ensure the best agreement between the calculations using the Schulman model and the Tsu-Esaki formula (see the inset in Figure 2).

The parameter $\Gamma_{n}^{*}$ has values orders of magnitude greater than $\Gamma_{n}$, and its evaluation is a non-trivial task, which is usually carried out on the basis of experimental data. This also applies to the remaining parameters of the model (including temperature parameters, i.e., thermal energy and coefficients before the components of the current density), since the Tsu-Esaki formula, which serves as a verification basis for the Schulman model, does not have a high degree of correspondence to the data of VAC measurements. Also, to increase the adequacy of the model, parasitic resistance, interelectron interaction and thermal current are included in the calculation [28,36,37]. The Schulman model for regression is determined by the following system of equations:

(10) $$\left\{\begin{array}{c} I\left(V_{D}\right)=I_{RT}\Gamma^{*}F\left(\frac{\varepsilon }{\Gamma^{*}}\right)\left(f_{2D}\left(\varepsilon \right)-f_{2D}\left(\varepsilon +qV_{D}\right)\right)+I_{T}\left(e^{\frac{V_{D}}{V_{T}}}-1\right)\\ \varepsilon =\varepsilon_{0}-k_{V}qV_{D}\\ F\left(\frac{\varepsilon }{\Gamma^{*}}\right)=\frac{1}{\pi }\mathrm{arctg}\frac{\varepsilon }{\Gamma^{*}}+\frac{1}{2}\\ f_{2D}\left(\varepsilon \right)=\ln\left(1+e^{\frac{E_{F}-E}{V_{RT}}}\right)\\ V=V_{D}-RI\left(V_{D}\right) \end{array}\right.$$

where $\left\{\varepsilon_{0},\Gamma^{*},k_{V},E_{F},I_{RT},V_{RT},I_{T},V_{T},R\right\}$—parameters determined from measurements.

However, the described Schulman model has a drawback that is critical for introducing additional factors into the model and estimating its parameters, namely, the occurrence of saturation current in the calculations associated with resonant tunneling.

To explain the occurrence of saturation current, let us consider the case when there is only one resonant level in the RTS quantum well. Then,

(11) $$J_{RT}\left(V\right)=\frac{q}{\hbar }\Gamma F\left(\varepsilon \right)\left(f_{2D}\left(\varepsilon \right)-f_{2D}\left(\varepsilon +qV\right)\right)$$

To prove the fact of the resonant tunneling current saturation, the authors consider the case of large external voltages V→∞. From some values of the external voltage V, let us assume that the difference between the Fermi level at the source and the energy of the resonant level is much greater than kT, and, as a consequence, in the argument of the natural logarithm of the function $f_{2D}\left(\varepsilon \right)$, it is obtained $exp\left(\left(E_{F}-\varepsilon \right)/kT\right)\gg 1$:

(12) $$f_{2D}\left(\varepsilon \right)=\frac{mkT}{2\pi \hbar^{2}}\ln\left(1+e^{\frac{E_{F}-\varepsilon }{kT}}\right)\approx \frac{mkT}{2\pi \hbar^{2}}\ln\left(e^{\frac{E_{F}-\varepsilon }{kT}}\right)=\frac{m}{2\pi \hbar^{2}}\left(E_{F}-\varepsilon \right)$$

Also, at V→∞, let us assume that the difference between the Fermi level in the drain $\left(E_{F}-qV\right)$ and the resonant level energy is sufficiently less than zero to neglect the contribution from the exponential in the argument of the natural logarithm of the function $f_{2D}\left(\varepsilon \right)$. The validity of this fact is obvious due to the lower decrease rate of the resonant level energy as a function of voltage $d\varepsilon /dV=-k_{V}q$ in comparison with the change rate of the Fermi level in the drain $\left(E_{F}-qV\right)$, equal to $d\left(E_{F}-qV\right)/dV=-q$. Therefore,

(13) $$f_{2D}\left(\varepsilon +qV\right)=\frac{mkT}{2\pi \hbar^{2}}\ln\left(1+e^{\frac{E_{F}-qV-\varepsilon }{kT}}\right)\approx \frac{mkT}{2\pi \hbar^{2}}\ln1=0$$

Substituting (12) and (13) into the expression for the resonant tunneling current density (9), the following expression is obtained:

(14) $$J_{RT}\left(V\right)\approx \frac{qm\Gamma }{2\pi \hbar^{3}}F\left(\varepsilon \right)\left(E_{F}-\varepsilon \right).$$

In relation (14), the function $F\left(\varepsilon \right)$ is expanded into a series. For this, let us use the properties of the arctangent and its expansion into a Maclaurin series for x→−∞.

(15) $$\mathrm{arctg}x=-\frac{\pi }{2}-\mathrm{arctg}\frac{1}{x}=-\frac{\pi }{2}-\frac{1}{x}+\frac{1}{3x^{3}}-\ldots$$

Considering that for V→−∞ the ratio ε/Γ→−∞, the function $F\left(\varepsilon \right)$ for ε/Γ→−∞ can be written as

(16) $$F\left(\varepsilon \right)=\frac{1}{\pi }\mathrm{arctg}\frac{\varepsilon }{\Gamma }+\frac{1}{2}=-\frac{\Gamma }{\pi \varepsilon }+\frac{1}{3\pi }{\left(\frac{\Gamma }{\varepsilon }\right)}^{3}-\ldots$$

Then, the following expression for the current density is obtained:

(17) $$J_{RT}\left(V\right)\approx \frac{qm\Gamma^{2}}{2\pi^{2}\hbar^{3}}-\frac{qm\Gamma E_{F}}{2\pi^{2}\hbar^{3}}\frac{\Gamma }{\varepsilon }-\frac{qm\Gamma^{2}}{6\pi^{2}\hbar^{3}}{\left(\frac{\Gamma }{\varepsilon }\right)}^{2}+\frac{qm\Gamma E_{F}}{6\pi^{2}\hbar^{3}}{\left(\frac{\Gamma }{\varepsilon }\right)}^{3}+\ldots$$

When ε/Γ→−∞ the highest powers of series (17) can be taken equal to zero, it follows that the current density when V→∞ is equal to

(18) $$J_{RT}\left(V\right)\approx \frac{qm\Gamma^{2}}{2\pi^{2}\hbar^{3}}$$

That is, in the case of increasing voltage, when the resonant level energy is less than the level of the conduction band bottom in the source, according to the Schulman model, the current reaches saturation. But, at such values of the resonant level energy in the source, there are no electrons that can potentially form a current; therefore, the resonant tunneling component should be equal to zero, which contradicts the prediction according to the Schulman model (11).

The saturation current is determined by the resonant level width; therefore, the problem of saturation current is aggravated by taking into account inelastic scattering, which makes an additional contribution to the resonant level width. At the same time, from the experience of modeling current transfer in RTS, it follows that the correct consideration of inelastic scattering is critically important for obtaining adequate results for the peak point of the RTD VAC. It should be noted that the currently used modifications of the Schulman model assume that the valley point of the RTD VAC and the curvature of the second region of differential conductivity are determined by the thermal current, but the presence of saturation current contradicts these ideas. Using the expression for the resonant tunneling current and the expression for the total current in the RTD leads to the fact that the resonant tunneling component affects the valley current and the curvature of the initial region, which complicates the determination of the model parameters, which, in turn, makes SPICE models based on the Schulman model difficult to use. Thus, the predicted saturation of the resonant tunneling current “blurs” the physical picture of charge transfer processes and lowers the validation level of the model.

It was shown above that the reason for the appearance of saturation current on the calculated RTD VAC is the representation of the tunnel transparency coefficient by Lorentzian functions. This problem required searching for

(19) $$T\left(E\right)=\sum_{n=1}^{N}\varphi \left(\frac{E-\varepsilon_{n}}{\Gamma_{n}}\right)$$

other functions of the same class as the members of the sum used in the Schulman model of the form

(20) $$\varphi^{Ш}\left(x\right)=\frac{1}{1+x^{2}}$$

For $\Gamma_{n}$ to have the meaning of the half-width of a resonant level, any resonant function must have the following property:

(21) $$\varphi \left(1\right)=\frac{1}{2}\varphi \left(0\right)$$

It is easy to verify that function (20) has this property.

The Lorentzian function is used for approximation as an ansatz obtained from the idea that the broadening profile of discrete states is related to the decay law of these states, which has an exponential form, via the Fourier transform [38]. However, it is pointed out that for small times the decay law of quantum states has a quadratic form, applying the Fourier transform to which an exponential decrease in the broadening profile is obtained with increasing distance from the discrete state energy [39]. Such asymptotic behavior is of a higher order than that of the Lorentzian function, so using a function with an exponential decrease in the profile could solve the saturation problem. An example of such a function is the inverse square hyperbolic cosine, which is used to describe tunneling through quantum dots [40].

Thus, to solve the problem of the resonant tunneling current density saturation, an odd, bounded, monotonically decreasing function with an asymptotic behavior of a higher order than (1) is proposed:

(22) $$\varphi^{k}\left(x\right)=\frac{1}{{\mathrm{ch}}^{2}\left(\kappa x\right)}$$

where the coefficient $\kappa =\mathrm{arch}\sqrt{2}$ is chosen to satisfy the condition (21).

Based on this, it is proposed to estimate the tunnel transparency coefficient as follows:

(23) $$T\left(E\right)=\sum_{n=1}^{N}\frac{1}{{\mathrm{ch}}^{2}\left(\kappa \frac{E-\varepsilon_{n}}{\Gamma_{n}}\right)}$$

Then, the function $F\left(\varepsilon_{n}\right)$ takes the form

(24) $$F^{*}\left(\varepsilon_{n}\right)=\frac{1}{\pi }\int_{-\frac{\varepsilon_{n}}{\Gamma_{n}}}^{\infty }\varphi^{k}\left(x\right)dx=\frac{1}{\pi }\int_{-\frac{\varepsilon_{n}}{\Gamma_{n}}}^{\infty }\frac{1}{{\mathrm{ch}}^{2}\left(\kappa x\right)}dx=\frac{1}{\pi \kappa }{\left.\mathrm{th}\kappa x\right|}_{-\frac{\varepsilon_{n}}{\Gamma_{n}}}^{\infty }=\frac{1}{\pi \kappa }\left(1+\mathrm{th}\left(\kappa \frac{\varepsilon_{n}}{\Gamma_{n}}\right)\right)$$

where the designation $F^{*}$ is introduced to distinguish the proposed model from the traditional one.

The function $F^{*}\left(\varepsilon_{n}\right)$ has an admitted region [0,2/πκ], unlike the function $F\left(\varepsilon_{n}\right)$, whose admitted region is [0,1]. In order for the domains of the functions $F\left(\varepsilon_{n}\right)$ and $F^{*}\left(\varepsilon_{n}\right)$ to coincide, let us redefine the function as

(25) $$F^{*}\left(\varepsilon_{n}\right)=\frac{1}{2}\left(1+\mathrm{th}\left(\kappa \frac{\varepsilon_{n}}{\Gamma_{n}}\right)\right)$$

The authors will aim to prove that abandoning the standard relation (8) for existing models in favor of (25) allows the problem of the resonant tunneling current saturation to be solved. The asymptotic behavior of the function $F^{*}\left(\varepsilon_{n}\right)$ is determined by the hyperbolic tangent function. When x→−∞,

(26) $$\mathrm{th}\left(x\right)=\frac{e^{2x}-1}{e^{2x}+1}=-1+2\frac{1}{e^{-2x}+1}\approx -1+2\frac{1}{e^{-2x}}=-1+2e^{2x}$$

Then, the function $F^{*}\left(\varepsilon \right)$ at ε/Γ→−∞ can be represented as

(27) $$F^{*}\left(\varepsilon \right)\approx \frac{2}{\pi \kappa }e^{2\kappa \frac{\varepsilon }{\Gamma }}$$

Since, taking into account L’Hôpital’s rule, it is true that

(28) $$\underset{x\to -\infty }{\lim}xe^{x}=\underset{x\to -\infty }{\lim}\frac{x}{e^{-x}}=-\underset{x\to -\infty }{\lim}\frac{1}{e^{-x}}=0$$

then the following expression is obtained for the resonant tunneling current density at ε/Γ→−∞:

(29) $$J_{RT}\left(V\right)\approx \frac{qm\Gamma }{\pi^{2}\hbar^{3}\kappa }e^{2\kappa \frac{\varepsilon }{\Gamma }}\left(E_{F}-\varepsilon \right)=\frac{qm\Gamma E_{F}}{\pi^{2}\hbar^{3}\kappa }e^{2\kappa \frac{\varepsilon }{\Gamma }}-\frac{qm\Gamma }{\pi^{2}\hbar^{3}\kappa }\varepsilon e^{2\kappa \frac{\varepsilon }{\Gamma }}\approx 0$$

**Figure 3:**
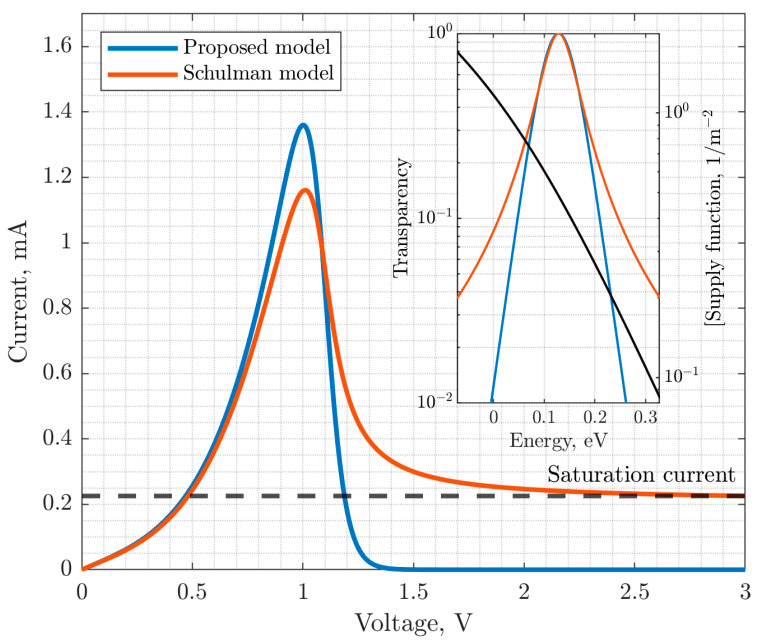
RTS VAC and tunnel transparency coefficient (on inset) according to the Schulman model and the proposed model.

Thereby, when using the function $F^{*}\left(\varepsilon_{n}\right)$ in the calculations of the resonant tunneling current density, the problem of current saturation does not arise. Figure 3 shows the graphs of the tunnel transparency coefficient, as well as the RTS VAC, calculated using the Schulman model and the proposed function for the tunnel transparency coefficient, which does not saturate the resonant-tunneling component of the current in the RTD.

Thereby, the proposed function $F^{*}\left(\varepsilon_{n}\right)$ is based on the hyperbolic cosine for estimating the tunnel transparency coefficient. It allows the problem of the traditional approach to describing the resonant nature of transparency using Lorentzian curves to be solved, which leads to non-physical current saturation in the negative differential conductivity region. The problem of the fundamental impossibility of adequately modeling the contrast of the negative differential conductivity region of the RTD VAC within the existing compact models is solved.

## 3. Results

**Figure 4:**
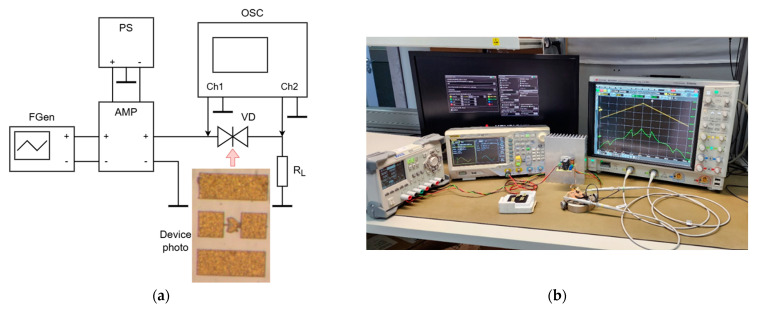
Schematic diagram and photo of chip with RTD device (**a**); photo of the setup for measuring the RTD VAC (**b**); Legend: FGen—RIGOL DG4102 functional generator; AMP—audio frequency amplifier based on the TDA7293 integrated circuit; OSC—Keysight MSOS804A oscilloscope; PS—RIGOL DP832 power supply; R_L_—10 Ohm load resistance; VD—RTD under test (DUT).

To validate the developed model, the following RTDs were simulated and the calculation results were compared with the VAC measurement results. The following type of RTDs served as experimental samples: AlAs with barrier thickness of 2.83 nm and 4.53 nm; GaAs with a well thickness of 3.95 nm; spacers with a thickness of 2.26 nm; transition layers with a thickness from 30 to 1500 nm with gradient doping from 7 × 10^16^ cm^−3^ with a mesa diameter of 10 μm; the structure was grown in the (100) direction. A sawtooth generator and an oscilloscope were used to measure the VAC; the connection diagram of the RTD sample and a photo of the setup are shown in Figure 4.

The experimental study of the RTD VAC is based on the technique of measuring the VAC of nonlinear elements, which implies the simultaneous measurement of the instantaneous voltage value on the element under study (VD) and on the series load resistance (RL) under the action of alternating voltage at the input of the VD-RL circuit. The FGen functional generator was used as an alternating voltage source, which generated a sawtooth-shaped probing alternating voltage with a frequency of $F_{G}=10\;kHz$. To reduce the output resistance of the alternating voltage source at the input of the VD-RL circuit, an AMP amplifier with a power of 140 W was used, which was powered from a PS power source with an output voltage of ±30 V. The output resistance of the amplifier was 4 Ohm. Thus, the FGen and AMP circuit can be considered an ideal alternating voltage source, the output resistance of which can be neglected when calculating the parameters of the RTD VAC.

Thus, the instantaneous current through the diode VD can be defined as

(30) $$i_{VD}\left(t\right)=\frac{u_{Ch2}\left(t\right)}{R_{L}}$$

The voltage across the diode at any given moment can be determined as

(31) $$u_{VD}\left(t\right)=u_{Ch1}\left(t\right)-u_{Ch2}\left(t\right)$$

The error estimate of the hysteresis measurement consists, firstly, of the measurement time scale accuracy of $u_{Ch1}\left(t\right)$ and $u_{Ch2}(t)$, which is determined by the number of sampling points per period of the probing sawtooth voltage. In the experiments carried out, it was 2048 points per period. Due to the relatively large period of the probing sawtooth voltage *T*, the jitters of the FGen generator and the OSC oscilloscope, which are about 10^−9^ s and 10^−13^ s, respectively, can be neglected.

Secondly, the error is determined by the noise of the generator FGen, the amplifier AMP, and also the oscilloscope’s self-noise.

The deviation of the generator’s effective voltage caused by harmonic distortions at an output voltage of 1 V_p-p_, in accordance with the technical description, (RMS) is no more than $∆U_{G}=2$ mV.

The value of the AMP amplifier’s self-noise, neglecting shot noise and flicker noise, can be estimated as the product of the effective value of thermal noise at the amplifier input with a resistance $R_{in}=$10 kOhm in a band B=1 MHz (limited by the oscilloscope settings) at a temperature T=300 K and the AMP gain $G_{AMP}=27.6$. That is, $∆U_{AMP}=\sqrt{4kTR_{in}B}\times G_{AMP}\approx 0.5$ mV.

The oscilloscope’s self-noise when measuring in channel Ch1 with a vertical scale division value of 2 V/div is (RMS) $∆U_{Ch1}=$ 50 mv.

The oscilloscope’s self-noise when measuring in channel Ch2 with a vertical scale division value of 200 mV/div is (RMS) $∆U_{Ch2}=$ 6.4 mV.

In order to reduce the influence of the indicated errors on the results, averaging of measurements at each point $u_{Ch1}(t)$ and $u_{Ch2}(t)$ over *N* = 65,534 measurements was introduced.

Thus, according to the theory of processing indirect measurements, as well as the theory of statistical analysis of measurement results [28], the effective value of the total noise voltage, taking into account measurements in two oscilloscope channels, can be estimated as

(32) $$∆U=\sqrt{2{\left(∆U_{G}G_{AMP}\right)}^{2}+{2∆U_{AMP}}^{2}+{∆U_{Ch1}}^{2}+{∆U_{Ch2}}^{2}}/\sqrt{N}=0.36\;mV\;$$

Estimates of the resonant tunneling current parameters within the proposed “non-saturating” compact model and the Schulman model are given in Table 1.

**Figure 5:**
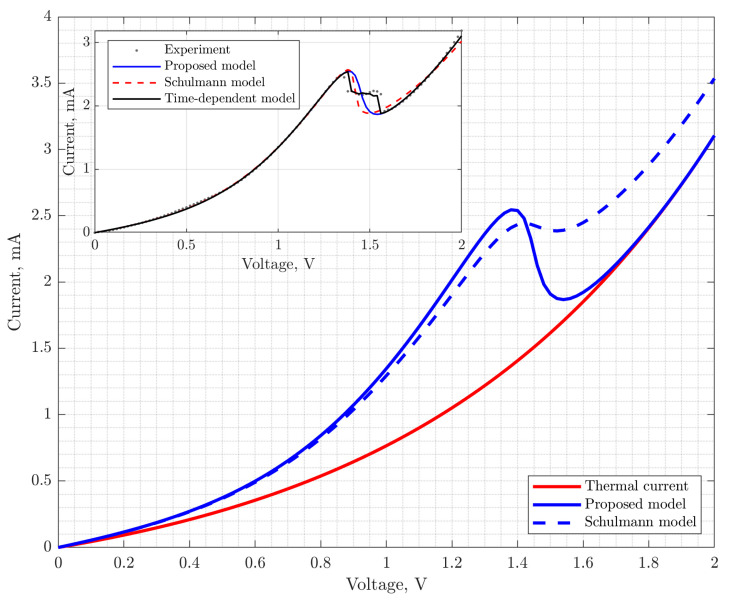
Experimental RTD VACs compared with simulation results. The inset shows the VAC of test RTDs and the thermal component of the current according to the proposed model.

The characteristic VACs of the RTD test samples in comparison with the calculation results are shown in Figure 5. Large axes show results of RTD’s VAC simulation with the Schulmann and proposed models with the same set of parameters (see Table 1, row 2). On the inset showing RTD’s VAC measurement are shown the simulation with the proposed model and best fitting with the Schulmann model (set of parameters given in Table 1, row 1). The results are discussed in the next section. Also, simulation results taking into account parasitic impedances with time-dependent equivalent circuit model are given on the inset. The equivalent circuit of RTD used in the simulations was a nonlinear element with VAC defined by a compact model with resistor and inductor in series and capacitor in parallel.

## 4. Discussion

As it can be seen from Table 1, the estimates of all parameters, except $\Gamma^{*}$, agree quite well for the two models considered. The discrepancy in the estimates of $\Gamma^{*}$ is explained by the need to level out the current saturation within the Schulman model in order to achieve good agreement in the second region of positive differential conductivity. Indeed, as has been shown, the saturation current is proportional to the square of $\Gamma^{*}$ (see Formula (18)), and for the Schulman model, $\Gamma^{*}$ is 4 times smaller than for the proposed model. Hence, it can be concluded that the same degree of correspondence between the calculation results using the Schulman model and the experiment can be achieved with other parameters $\Gamma^{*}$, $V_{T}$ and $I_{T}$, which indicates the ambiguity of the estimates of the Schulman model parameters. The identified ambiguity of the estimates also suggests that, within the Schulman model, the resonant-tunneling current component contributing exclusively to the first region affects the parameters of the thermal current, which determines the second region of differential conductivity, contradicting the ideas about current transfer in these regions of the RTD VAC. The proposed compact model of the RTD VAC is successfully validated in a wide voltage range, due to the fact that it is devoid of the listed problems of traditionally used models. The problem of non-physical saturation of the resonant-tunneling component of the current is successfully solved using a new function of the tunnel transparency coefficient profile, which does not lead to saturation due to the asymptotic behavior of a higher order. Also, computational effectiveness of the proposed model (same for Schulmann model) due to its compactness is significantly more than that of theoretical models, based on integral and differential equations, which requires computationally expensive numerical methods to solve, especially with self-consistent accounting of interelectronic interaction.

## 5. Conclusions

Therefore, the solution to the fundamental problem of assessing the functional nature of the diode channel transparency depending on the energy characteristics of the model (the problem of saturation of the resonant component of the current) in the traditional empirical model, as well as its transformation into a class of compact (see the publications of the authors [28,32]) predictor models based on perceptron expansion made it possible, firstly, to preserve the most important advantages of the traditional Schulman model with the ability to analyze the main VAC characteristics (curvature, contrast, etc. [28]) and artifacts in the negative differential conductivity region (plateau, hysteresis [32]), primarily due to the solution to the fundamental problem of non-physical current saturation, due to which traditional compact models provide less accurate estimates of the valley current and contrast of the negative differential conductivity region compared to the presented model (see Figure 5 and Table 1), even with the regression analysis. Secondly, the proposed model makes it possible to provide an effective solution to the synthesis problem, on the one hand, without labor-intensive quantum mechanical modeling algorithms, and, on the other hand, without losing the ability to physically interpret and analyze the physical processes that determine the RTD VAC.

## Figures and Tables

**Table 1:** Average values and RMS deviations of estimates of resonant tunneling current parameters.

	Parameter	ε, eV	$\Gamma^{*}$, meV	$k_{V}$	$V_{RT}$, eV	$I_{RT}$, mA
1	Schulman	1.20	23.17	0.82	0.23	0.75
2	Proposed model	1.33	92.72	0.91	0.24	0.73

## Data Availability

The original contributions presented in the study are included in the article, further inquiries can be directed to the corresponding author.
